# Atypical Superior Mesenteric Artery Syndrome in an Elderly Male Without Classic Risk Factors

**DOI:** 10.7759/cureus.99697

**Published:** 2025-12-20

**Authors:** Bhavik Singh, Zoë M Rushetsky, Talib Araim, Bhavi Purohit

**Affiliations:** 1 Department of Osteopathic Medicine Program, Philadelphia College of Osteopathic Medicine, Suwanee, USA; 2 Family Medicine/Hospital Medicine, Wellstar North Fulton Hospital, Roswell, USA

**Keywords:** duodenal obstruction, elderly patient, gastrojejunostomy tube complication, mesenteric fat loss, superior mesenteric artery syndrome

## Abstract

Superior mesenteric artery syndrome (SMAS) is a rare cause of proximal intestinal obstruction caused by compression of the distal third portion of the duodenum between the aorta and superior mesenteric artery (SMA) due to loss of mesenteric fat. This is typically seen in younger patients with rapid weight loss or spinal deformity correction. We report an atypical case of SMAS that occurred in an 80-year-old male who presented with progressive nausea, unintentional weight loss over six months, and vomiting. Despite gastrojejunostomy tube placement, symptoms persisted and were complicated by peritonitis as a result of tube migration. Imaging revealed an aortomesenteric distance of 4.2 mm, proximal duodenal dilation, and a narrowed aortomesenteric angle. All of which are consistent with SMAS. This was attributed to gradual mesenteric fat loss from chronic undernutrition. This case highlights that SMAS may occur in elderly patients without rapid weight loss, scoliosis correction, or prior surgery and may present as a chronic rather than acute condition. Clinicians should maintain a high index of suspicion in older adults with unexplained vomiting and gradual weight loss to allow for early diagnosis, prevent procedural complications, and optimize nutritional recovery.

## Introduction

SMAS is a rare cause of proximal intestinal obstruction, typically resulting from compression of the third portion of the duodenum between the aorta and the overlying SMA due to loss of mesenteric fat [1]. Rapid weight loss depletes visceral fat stores, eliminating the fat pad that typically occupies the space between the SMA and the third portion of the duodenum [1,2].

The aorticomesenteric angle, defined as the angle formed between the abdominal aorta and the SMA as it branches anteriorly, normally measures greater than 22°. In SMAS, objective diagnostic criteria include an aorticomesenteric angle ≤22° and the aorticomesenteric distance ≤8 mm, both of which reflect clinically significant vascular compression of the duodenum [1,2]. 

SMAS is typically seen in younger individuals who experience rapid weight loss, eating disorders, or spinal deformity correction [3]. Although no ethnic predilection has been identified, women are affected more frequently. This is thought to be due to a lower baseline body mass index (BMI) and a higher prevalence of eating disorders rather than any anatomical differences [1,4].

Because of the rarity of SMAS, with an estimated incidence of only 0.01%-0.3% [4,5], most literature consists of single-case reports and small series. We describe an atypical presentation of SMAS in an elderly man without traditional risk factors to remind clinicians that this diagnosis can occur outside the usual demographic and may present as a chronic rather than an acute condition.

## Case presentation

An 80-year-old male with coronary artery disease (two prior myocardial infarctions, stent placement, and angioplasty in the 1990s), chronic kidney disease stage 3b, gastroesophageal reflux disease, and prior cervical spinal surgery with titanium rod placement presented with progressive gastrointestinal symptoms beginning in June 2025. 

A review of the preceding six months revealed an unintentional weight loss of about 24 lbs, decreasing from 174 lbs to 150 lbs, and a corresponding drop in BMI from 21.7 to 18.7. His waist circumference decreased from 36 to 33 inches. He reported a marked reduction in appetite, such as being able to consume only half of a burger, where he previously ate two. Despite this, he denied dizziness, depression, or functional decline and remained able to perform activities of daily living independently.

Additional history revealed chronic constipation managed with stool softeners, including senna and docusate sodium. He also reported intermittent black stools and an episode of bright red rectal bleeding prior to the initial hospitalization. Additionally, his last colonoscopy, done 10 years ago, was normal. Although he quit smoking in 1981, he resumed nicotine use through vaping about three years ago. He also reported a daily intake of roughly one shot of vodka. There was no history of scoliosis, gastric surgery, psychiatric illness, or eating disorders documented. 

In June 2025, he left a social gathering early due to sudden lightheadedness, followed by nausea and multiple episodes of coffee-ground emesis throughout the night. The following morning, he was emergently transported to the hospital, where laboratory evaluation revealed acute upper gastrointestinal bleeding with symptomatic anemia, necessitating blood transfusions. Five days later, he was discharged without a definitive diagnosis documented during that initial admission.

Two weeks after the initial ER visit, the patient presented to the emergency department again, following a second episode of intractable vomiting, which he initially attributed to possible food poisoning. However, this was ruled as an unlikely cause since the individuals who had shared the same meal remained asymptomatic. During this second admission, he underwent evaluation for gastrointestinal bleeding and a decreased tolerance of oral intake, after which a gastrojejunostomy (GJ) tube was placed. Two weeks after the procedure, the GJ tube migrated out of the stomach, causing severe abdominal pain from peritonitis caused by leakage of gastric contents into the peritoneal cavity. This complication was most likely attributable to malposition of the tube secondary to the patient’s posteriorly displaced stomach, requiring GJ tube repositioning. Additionally, the peritonitis was treated with intravenous piperacillin-tazobactam and supportive care.

**Figure 1:**
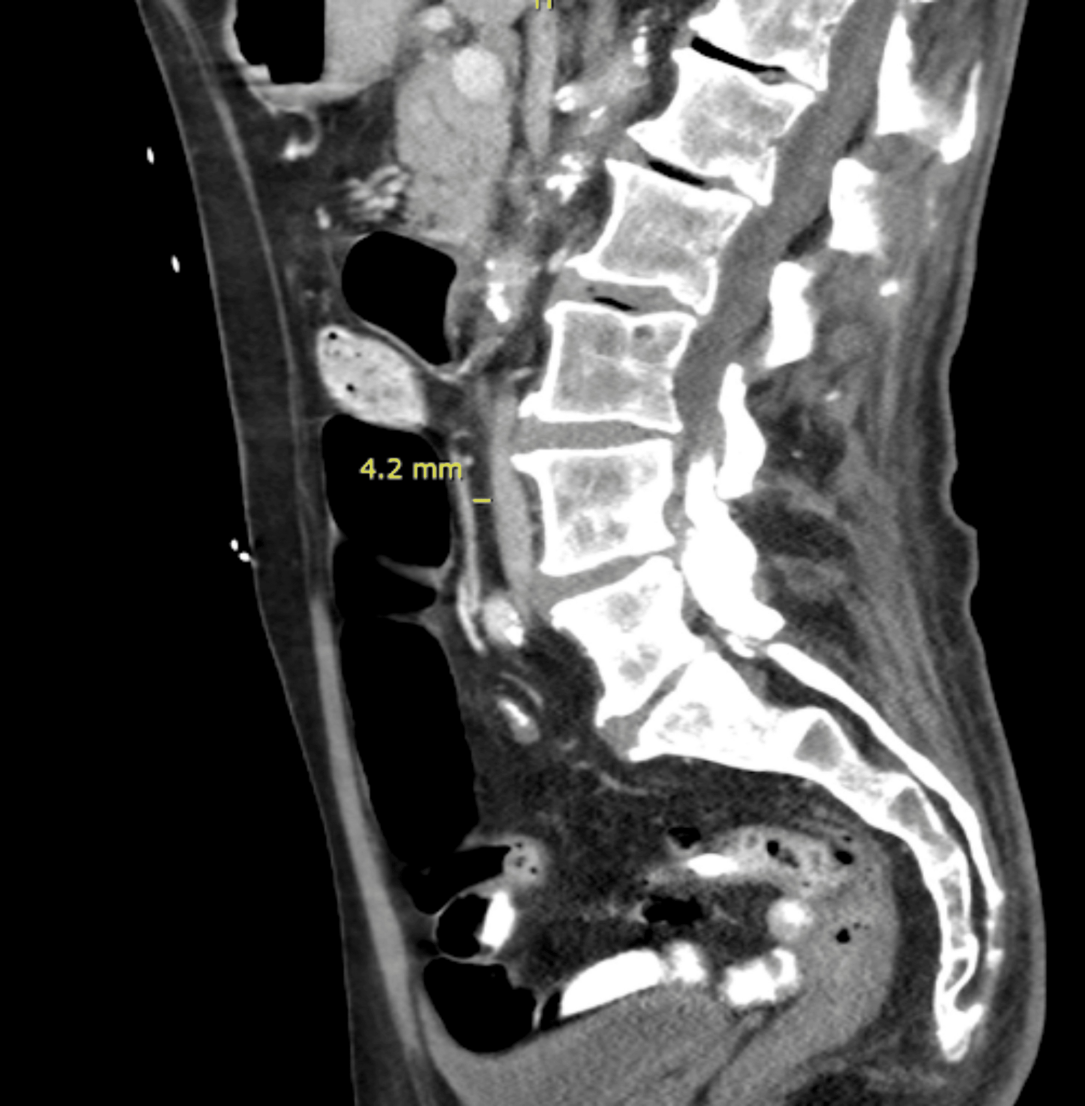
Sagittal contrast-enhanced CT image of the abdomen. Contrast-enhanced sagittal CT image of the abdomen demonstrating compression of the third portion of the duodenum between the SMA and the aorta. The measured aortomesenteric distance is 4.2 mm (yellow line).

**Figure 2:**
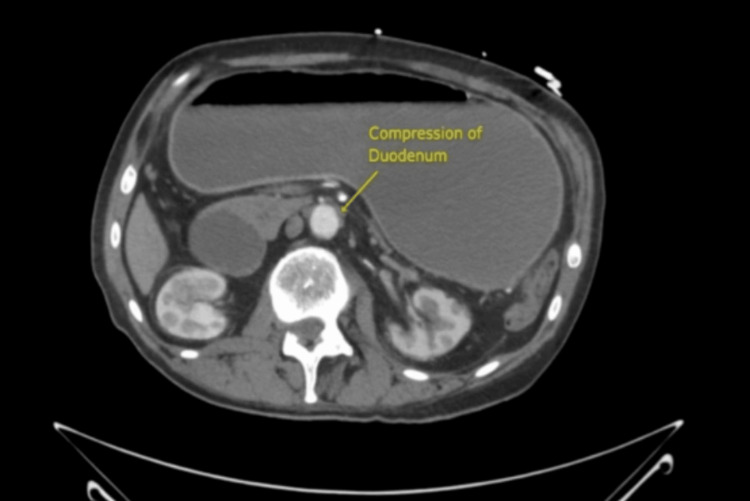
Axial contrast-enhanced CT image of the abdomen. Contrast-enhanced axial CT image demonstrating compression of the third portion of the duodenum between the SMA and the abdominal aorta (shown in yellow).

**Figure 3:**
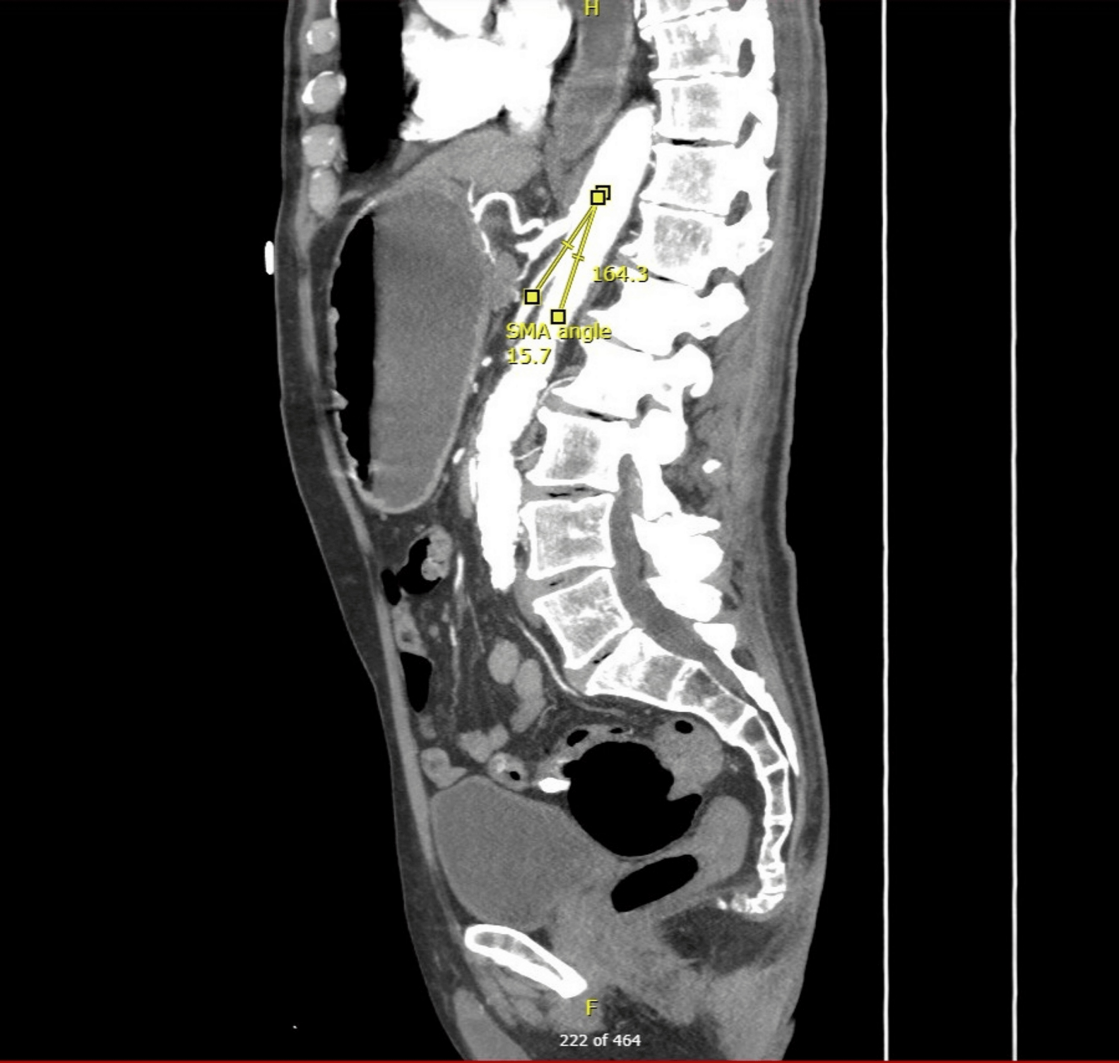
Sagittal contrast-enhanced CT image of the abdomen. Contrast-enhanced sagittal CT scan showing a markedly decreased aortomesenteric angle of 15.7°. Measurement lines demonstrate the acute narrowing of the aortomesenteric angle, compressing the third portion of the duodenum.

On physical examination, he appeared thin but not cachectic. Computed tomography (CT) imaging showed compression of the third portion of the duodenum between the aorta and the SMA, with an aortomesenteric distance of 4.2 mm (Figure 1), proximal duodenal dilation (Figure 2), and a narrowed aortomesenteric angle of 15.7° (Figure 3).

Following the confirmed diagnosis of SMAS, the patient underwent conservative management consisting of nutritional rehabilitation with high-calorie supplementation, gradual advancement of enteral feeding, and correction of electrolyte abnormalities. Surgical intervention was deferred because his symptoms improved with nutritional recovery. Over the subsequent weeks, he demonstrated increased oral intake, weight stabilization, and reduction of postprandial discomfort. At follow-up, he reported resolution of vomiting and improved tolerance of meals, with plans for continued outpatient nutritional monitoring.

## Discussion

This case illustrates an atypical presentation of SMAS in an elderly male without the classic risk factors of recent spinal surgery, eating disorders, or abrupt weight loss. The atypical presentation in this patient was the gradual development of SMAS in the setting of chronic undernutrition rather than an acute, identifiable precipitant. This demonstrates that progressive loss of mesenteric fat can occur over time, even in the absence of overt cachexia, and may ultimately result in clinically significant duodenal compression.

SMAS is most often described as an acute condition that typically develops over days to several weeks following rapid weight loss or surgical intervention. However, reports show more indolent presentations with symptoms developing over months rather than weeks [1]. In this patient, gastrointestinal symptoms progressed over approximately six months. This slower trajectory reinforces the need to consider SMAS in patients with chronic, unexplained nausea, vomiting, and weight loss, particularly when standard evaluations fail to identify an alternative cause.

Although this patient did not exhibit classic precipitants, aspects of his clinical history may help explain the delayed diagnosis. Intermittent black stools and an episode of bright red rectal bleeding reasonably raised concern for gastrointestinal bleeding [6]. This may have directed early evaluation toward hemorrhagic etiologies rather than a mechanical obstruction. In addition, his longstanding constipation and reliance on stool softeners suggest reduced oral intake and possible chronic gastrointestinal dysfunction [7]. While causality cannot be established, these overlapping clinical features likely contributed to a gradual nutritional decline and obscured recognition of an underlying obstructive process. This case highlights how competing symptoms can delay the diagnosis of chronic SMAS, particularly in older adults.

The most plausible mechanism of obstruction in this patient was progressive depletion of mesenteric fat due to chronic undernutrition, resulting in narrowing of both the aortomesenteric angle and distance, with sustained compression of the third portion of the duodenum. The following episode of peritonitis following gastrojejunostomy tube migration emphasizes the potential consequences of delayed diagnosis and altered anatomy in SMAS. 

Management of SMAS remains variable and is guided by symptom severity and underlying etiology, but is generally approached in a stepwise fashion [8]. Initial treatment focuses on conservative measures, including nutritional rehabilitation, small, frequent meals, and correction of electrolyte abnormalities, with escalation to parenteral nutrition or surgical intervention in refractory cases [4,9,10]. Surgical options, such as duodenojejunostomy, gastrojejunostomy, or division of the ligament of Treitz, aim to bypass or relieve the obstruction [4,11]. While earlier literature favored early operative management, more recent evidence supports an initial trial of conservative therapy whenever feasible [11]. In this patient, symptoms improved with nutritional optimization alone, allowing surgery to be deferred.

Overall, this case emphasizes that SMAS can occur outside of its traditional demographic profile and may present as a chronic condition in elderly patients with gradual weight loss. Maintaining a high index of suspicion in this population may allow for earlier diagnosis, reduce the risk of procedural complications, and support safer nutritional recovery.

## Conclusions

SMAS is classically associated with young women who have undergone spinal surgery or experienced rapid weight loss. This typically results in postprandial epigastric pain, which can further worsen nutritional status and lead to progressive clinical decomposition. This case demonstrates that SMAS can also result from gradual mesenteric fat atrophy due to chronic undernutrition in older men. By presenting an elderly patient with slowly progressive, nonspecific symptoms, this report expands clinician awareness of SMAS beyond its traditional risk groups. Early recognition, along with prompt nutritional and multidisciplinary management, is essential to prevent morbidity and improve outcomes. 

Given the unusual presentation similar to our case, SMAS may not originally be considered as a sequela of chronic undernutrition. It is vital that clinicians maintain a broad differential when evaluating unexplained vomiting and weight loss in older adults, as delayed recognition can lead to unnecessary interventions and complications.

## References

[1] Ganss A, Rampado S, Savarino E (2019). Superior mesenteric artery syndrome: a prospective study in a single institution. J Gastrointest Surg.

[2] Roy A, Gisel JJ, Roy V, Bouras EP (2005). Superior mesenteric artery (Wilkie's) syndrome as a result of cardiac cachexia. J Gen Intern Med.

[3] Pottorf BJ, Husain FA, Hollis HW Jr, Lin E (2014). Laparoscopic management of duodenal obstruction resulting from superior mesenteric artery syndrome. JAMA Surg.

[4] Biank V, Werlin S (2006). Superior mesenteric artery syndrome in children: a 20-year experience. J Pediatr Gastroenterol Nutr.

[5] Aw AE, Lee JW, Tan IJ, Ng CW (2022). Superior mesenteric artery syndrome: An unusual cause of abdominal compartment syndrome and bilateral lower limb ischemia. BJR Case Rep.

[6] Sabry AO, Sood T (2023). Rectal Bleeding. StatPearls [Internet].

[7] Liu LW (2011). Chronic constipation: current treatment options. Can J Gastroenterol.

[8] Wang YH, Takada T (1984). Superior mesenteric artery syndrome: report of four cases. Gastroenterol Jpn.

[9] Baltazar U, Dunn J, Floresguerra C (2000). Superior mesenteric artery syndrome: an uncommon cause of intestinal obstruction. South Med J.

[10] Balmaseda MT Jr, Gordon C, Cunningham ML, Clairmont AC (1987). Superior mesenteric artery syndrome after resection of an arteriovenous malformation in the cervical cord. Am J Gastroenterol.

[11] Burrington JD, Wayne ER (1974). Obstruction of the duodenum by the superior mesenteric artery--does it exist in children?. J Pediatr Surg.

